# Photochemical internalisation of chemotherapy potentiates killing of multidrug-resistant breast and bladder cancer cells

**DOI:** 10.1038/sj.bjc.6603895

**Published:** 2007-07-31

**Authors:** D K Adigbli, D G G Wilson, N Farooqui, E Sousi, P Risley, I Taylor, A J MacRobert, M Loizidou

**Affiliations:** 1 Department of Surgery, Royal Free and University College Medical School, UCL, London, UK

**Keywords:** photochemical internalisation, multidrug resistance, breast and bladder cancer, hypericin, photodynamic therapy

## Abstract

Multidrug resistance (MDR) is the major confounding factor in adjuvant solid tumour chemotherapy. Increasing intracellular amounts of chemotherapeutics to circumvent MDR may be achieved by a novel delivery method, photochemical internalisation (PCI). PCI consists of the co-administration of drug and photosensitiser; upon light activation the latter induces intracellular release of organelle-bound drug. We investigated whether co-administration of hypericin (photosensitiser) with mitoxantrone (MTZ, chemotherapeutic) plus illumination potentiates cytotoxicity in MDR cancer cells. We mapped the extent of intracellular co-localisation of drug/photosensitiser. We determined whether PCI altered drug-excreting efflux pump P-glycoprotein (Pgp) expression or function in MDR cells. Bladder and breast cancer cells and their Pgp-overexpressing MDR subclones (MGHU1, MGHU1/R, MCF-7, MCF-7/R) were given hypericin/MTZ combinations, with/without blue-light illumination. Pilot experiments determined appropriate sublethal doses for each. Viability was determined by the 3-[4,5-dimethylthiazolyl]-2,5-diphenyltetrazolium bromide assay. Intracellular localisation was mapped by confocal microscopy. Pgp expression was detected by immunofluorescence and Pgp function investigated by Rhodamine123 efflux on confocal microscopy. MTZ alone (0.1–0.2 *μ*g ml^−1^) killed up to 89% of drug-sensitive cells; MDR cells exhibited less cytotoxicity (6–28%). Hypericin (0.1–0.2 *μ*M) effects were similar for all cells; light illumination caused none or minimal toxicity. In combination, MTZ /hypericin plus illumination, potentiated MDR cell killing, *vs* hypericin or MTZ alone. (MGHU1/R: 38.65 and 36.63% increase, *P*<0.05; MCF-7/R: 80.2 and 46.1% increase, *P*<0.001). Illumination of combined MTZ/hypericin increased killing by 28.15% (*P*<0.05 MGHU1/R) compared to dark controls. Intracytoplasmic vesicular co-localisation of MTZ/hypericin was evident before illumination and at serial times post-illumination. MTZ was always found in sensitive cell nuclei, but not in dark resistant cell nuclei. In illuminated resistant cells there was some mobilisation of MTZ into the nucleus. Pgp expression remained unchanged, regardless of drug exposure. Pgp efflux was blocked by the Pgp inhibitor verapamil (positive control) but not impeded by hypericin. The increased killing of MDR cancer cells demonstrated is consistent with PCI. PCI is a promising technique for enhancing treatment efficacy.

Resistance to multiple antineoplastic chemotherapeutic agents restricts the efficacy of cancer therapy (Leslie *et al*, 2005). Cancer progression is commonly associated with the formation of subcolonies within the heterogeneous tumour. Dependent on selection pressures, only a phenotypically elite subpopulation will survive. Exposure to chemotherapeutic drugs is an example of such a selection pressure from which only some of the tumour cells will survive by expressing a multidrug-resistant (MDR) phenotype, which renders them refractive to treatment. Two underlying mechanisms are (1) the overexpression of P-glycoprotein (Pgp) efflux pumps that eject drugs from the cytosol and (2) the action of lysosomes in degrading drugs taken up by endosomes.

A well-studied mechanism for MDR is the decrease in cellular drug accumulation mediated by the ATP-binding cassette (ABC) family of transporters. ABC transporters, for example, Pgp membrane efflux pumps, are expressed in normal tissues including intestinal epithelia, liver, renal tubules, and lung, where they mediate the efflux of toxins and drugs (Han and Zhang, 2004; Leslie *et al*, 2005). Transporter overexpression by a colony of tumour cells causes the expulsion from the cytoplasm of anti-cancer drugs before they reach their site of action and results in cellular and clinical MDR. Although initially attributed only to Pgp upregulation (Ambudkar *et al*, 1999), MDR may result from various overexpressed ABC transporters, eg, cancer-resistance protein and MDR-associated proteins (Shepard *et al*, 2003; Lepper *et al*, 2005).

A number of macromolecular and low molecular weight drugs do not bind cell surface receptors but are targeted to intracellular compartments. Often unable to freely diffuse across lipid membranes, drugs are taken up by endocytosis before they can be released intracellularly to reach their targets. Endocytosed drugs may be degraded in lysosomes and this stops their biological activity. One MDR mechanism in cancer is the upregulation of this process to significantly limit drug efficacy (Høgset *et al*, 2004).

Photochemical internalisation (PCI) is an extension of photodynamic therapy. The principle of PCI is to localise the photosensitiser together with the drug or gene of choice in endocytic vesicles within target cells, with the photosensitiser specifically localising to the vesicular membrane (Høgset *et al*, 2004; Ndoye *et al*, 2004; Dietze *et al*, 2005). Irradiation of cells with light of a specific wavelength will excite the photosensitiser to produce reactive oxygen species which subsequently rapture the membranes (Figure 1), resulting in drugs release. (Høgset *et al*, 2004; Ndoye *et al*, 2004; Dietze *et al*, 2005). Consequently, PCI enables a more efficient delivery of drug to their intracellular targets (Berg *et al*, 1999; Selbo *et al*, 2001; Høgset *et al*, 2004). In cancer treatment, PCI as a more selective procedure than systemic chemotherapy will have reduced systemic effects and its activity can be restricted to irradiated tissues.

PCI has been shown to potentiate the biological activity of various macromolecules (Berg *et al*, 2003), eg, the type I ribosome-inactivating protein Gelonin (Barbieri *et al*, 1993). This single-chain polypeptide cannot bind to the cell surface nor facilitate its own release from endocytic vesicles (Stirpe *et al*, 1980). Although a powerful inhibitor of protein synthesis in cell-free systems, it has low toxicity in intact cells and or *in vivo* (Barbieri and Stirpe, 1982; Scott *et al*, 1987). Gelonin localised in the same intracellular compartments as the photosensitiser disulphonated aluminium phthalocyanine (Selbo *et al*, 2000); the latter has been shown to localise within endocytic vesicles both *in vitro* (Moan *et al*, 1989) and *in vivo* (Høgset *et al*, 2004). Illumination of cells containing the two agents resulted in drug release into the cytoplasm (Høgset *et al*, 2004). PCI of gelonin resulted in a 300-fold reduction of protein synthesis compared to exposure to gelonin alone or light plus photosensitiser (PDT) alone.

Glucosylated polyethylenimine (PEI) in conjunction with PCI enhanced nonviral-mediated transfer of *wt-p53* into *p53*-deleted PANC3 (pancreas carcinoma) and *p53*-mutated FaDu (pharynx carcinoma) lines. PCI resulted in increased p53 mRNA expression by 2.3-fold in PANC3 cells, compared to PEI alone (Ndoye *et al*, 2004). PCI also increased apoptosis in both lines to levels similar to those achieved with chemotherapy. Thus by facilitating the release of wild-type *p53* from endocytic vesicles, PCI enhanced both the expression of *p53* and its activity (spontaneous apoptosis) compared to PEI alone in human cancer cell lines. The findings support PCI as an alternative procedure for gene delivery into the cytosol of target cells (Berg *et al*, 1999).

In a previous study involving our department, the photosensitiser disulphonated meso-tetraphenylporphyrin (TPPS_2a_) was co-administered with the anticancer drug doxorubicin, to a drug-sensitive breast cancer cell line and its resistant counterpart (MCF-7/S, MCF-7/R). Doses used for both agents were those that resulted in 50% cellular cytotoxicity. Strikingly, the administration and irradiation of TPPS_2a_, followed by doxorubicin incubation, resulted in significant increased killing for the MDR cells. Following confocal microscopy tracking, the authors suggested that this was due to the fact that endolysosomal vesicles which had incorporated TPPS_2a_ were destroyed upon illumination and therefore did not trap the drug doxorubicin (Lou *et al*, 2006).

We therefore decided to explore the use of PCI in circumventing drug resistance in two pairs of human cancer cell lines, comprising both drug-sensitive parental lines and MDR subclones, specifically the bladder and breast cancer cell lines MGHU1/S and MGHU1/R, and MCF-7/S and MCF-7/R. We also decided to investigate cytotoxic effects of sublethal concentrations of drugs, using the chemotherapeutic mitoxantrone (MTZ) and as photosensitiser the St John's Wort extract hypericin. The anthraquinone, hypericin, is synthesised by the *hypericum* genus of plants and it is probably the most powerful photosensitiser found in nature. Hypericin exhibits the characteristics that are fundamental for the application of PDT, including bright fluorescence, a high singlet oxygen quantum yield upon illumination and minimal dark toxicity (Agostinis *et al*, 2002). Previous PDT studies on the effect of hypericin on cultured fibroblasts or murine keratinocytes have shown concentration- and light-dependent phototoxicity (Yu *et al*, 1996; Theodossiou *et al*, 2004). Recently, an additional hypericin phototoxic mechanism against mitochondria has been identified. This does not depend on glutathione/H_2_O_2_ homeostasis (Theodossiou *et al*, 2006). Hypericin has also been shown to induce PDT in cancer cells *in vitro* (Assefa *et al*, 1999). However, no studies to date have investigated hypericin within the context of PCI.

We first determined the relative effect of hypericin-PDT and secondly we examined whether PCI using low dose hypericin was able to potentiate the cytotoxicity of MTZ on these cell lines.

## MATERIALS AND METHODS

### Cell culture preparation

Two sets of human cancer cell lines were used: (1) bladder cancer MGHU1/S (Masters *et al*, 1986) and its MDR counterpart MGHU1/R (grown in increasing adriamycin (ADR) concentrations, Govern *et al*, 1988); (2) breast cancer MCF-7/S (European Collection of Animal Cell Cultures, Porton Down, UK) and its MDR counterpart MCF-7/R. All, but MCF-7, were donated by Professor Masters, Prostate Cancer Research Centre, UCL. We previously showed Pgp overexpression by MGHU1/R and MCF-7/R (Davies *et al*, 1999). Cells were routinely grown in Dulbecco's modified Eagle's medium (DMEM) with 10% (*v/v*) fetal calf serum (FCS) and 0.1% (*v/v*) gentamycin, at 37°C. Cells were trypsinised (1 mg ml^−1^ in 0.2% phosphate-buffered saline (PBS)/EDTA), centrifuged (× 2, 400 **g**, 5 min) and used for passage or further experimental work. For plating into 96-well plates (100 *μ*l well^−1^), cells were re-suspended at 100 000 cells/100 *μ*l (MGHU1/S&R) or 200 000 cells/100 *μ*l (MCF-7/S&R). The different plating numbers reflected growth rates and ensured equivalent growth. (Reagents from Sigma, Dorset, UK, unless stated otherwise).

### Mitoxantrone and hypericin dose–response curves

#### Mitoxantone

Concentrations to be used in later combination experiments were determined by exposing cells to MTZ concentrations from 0.1 to 2 *μ*g ml^−1^. Cells were seeded in 96 well plates (see Cell culture preparation) for 24 h, incubated with MTZ for a further 24 h at 37°C, in a humidified 5% CO_2_ atmosphere and allowed to recover for 24 h. Wells were washed twice (PBS) after drug exposure and waste was removed using a suction pump at each stage. Viability was assayed by the MTT assay.

#### Hypericin

Experimental conditions for hypericin concentrations and illumination time for further combination experiments were determined as follows.

#### Time of hypericin exposure

Cells were exposed to hypericin for 4 h, which is sufficient time for the agent to be internalised (Theodossiou *et al*, 2004).

#### Dose response

**Figure 2:**
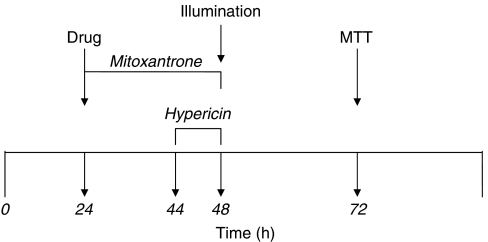
Experimental timeline for PCI on cells. PBS saline washes were performed after each drug incubation to ensure drug removal from cell cultures.

Cells were plated as described in Figure 2, wrapped in aluminium foil and allowed to settle and grow for 24 h at 37°C, 5% CO_2_. At this time, media in the wells was replaced with fresh media for a further 20 h and then media was again replaced with hypericin at 0.1, 0.2, 0.4, or 1.0 *μ*M for 4 h at 37°C, 5% CO_2_. Cells were washed and plates were illuminated for 1, 2, or 5 min. The cells were allowed to recover for a further 24 h, washed in PBS and assayed by MTT (Figure 2). All experimental steps, apart from illumination, were carried out in the dark to avoid inappropriate hypericin photoactivation; plates were wrapped in aluminium foil while in the incubator and lights were off in the laminar flow cabinets during cell work. The ‘dark’ control plates were never illuminated or exposed to light.

### Combination experiments

Combination experiments were carried out under dark conditions (see Mitoxantrone and hypericin dose-response curves). Cells were plated in 96-well plates for 24 h, 37°C, 5% CO_2_, at which point media was removed and replaced with one of the following: (1) MTZ alone for 24 h; (2) MTZ for 20 h, followed by washing (2 × PBS) and incubation with MTZ/hypericin containing media for 4 h; (3) fresh media for 20 h followed by hypericin containing media for 4 h; and (4) media alone in control wells. All non-combination wells were washed at 20 h and media containing appropriate agents replaced, to control for media change and fresh agents in the combination group (Figure 2). At the end of the 24 h incubation, cells were thoroughly washed and fresh media was replaced in the wells. Plates were illuminated for 2 min with blue light, Lumisource (maximum output at 420 nm 7 mW cm^−2^; PCI Biotech, Oslo, Norway) (120 s × 7 mW cm^−2^ equivalent to 840mJ cm^−2^), and subsequently incubated for a further 24 h, 37°C, 5% CO_2_. Viability was assessed using MTT. Drug concentrations used were 0.1 and 0.2 *μ*g ml^−1^ MTZ, 0.1 *μ*M and 0.2 *μ*M hypericin, alone, or in combinations. Control ‘dark’ plates were neither exposed to light nor illuminated. Control ‘normal light’ plates were not covered in aluminium foil or prepared in the dark or illuminated.

### MTT assay

The 3-[4,5-dimethylthiazolyl]-2,5-diphenyltetrazolium bromide (MTT) assay was used to determine cellular viability. Cell survival is quantified by assessing mitochondrial activity, that is, the reduction by mitochondrial dehydrogenases of the hydrophilic tetrazonium salt to a purple, insoluble formazan derivative. The latter's density is quantified by its absorption of visible light (570 nm). MTT (1 mg ml^−1^) was added to each well (100 *μ*l) and cells incubated for 3 h, 37°C, 5% CO_2_ (Uehlinger *et al*, 2000). MTT was replaced with dimethyl sulphoxide (100 *μ*l/well) to dissolve formazan crystals. Absorption was read on the plate reader (MR 700 Dynatech; 570 nm bandpass filter).

### Hypericin cellular uptake

The four cell lines were plated as described. After incubation (24 h, 37°C, 5% CO_2_), the medium was replaced and cells were incubated for another 20 h. At this time the medium was replaced with 1 *μ*M hypericin (100 *μ*l/well) and re-incubated for 4 h. Hypericin was washed off, replaced with PBS and intracellular hypericin fluorescence measured using a fluorescence reader (LS50B Perkin-Elmer fluorescence spectrometer, Beaconsfield, UK).

### Protocol for imaging experiments using MTZ and hypericin

Pgp expression (see Immunofluorescence staining for Pgp expression) and the intracellular localisation of MTZ and hypericin were investigated by growing cells on glass for microscopic visualisation. Cells were grown on 13 mm glass coverslips in 24-well plates (100 000/100 *μ*l for MCF-7, MCF-7/R and 50 000/100 *μ*l for MGHU1/S, MGHU1/R; 37°C, 5% CO_2_). After settling for 24 h, cells were designated to receive MTZ, hypericin, or a combination of the two. The incubation times followed are described in 2.5. The concentrations used were 1 or 2 *μ*g ml^−1^ MTZ and 1 or 2 *μ*M hypericin either singly or in combination. After drug incubation (24 h), cells were washed and illuminated for 2–4 min to ensure full photoactivation and then replaced in the incubator for 1, 4, 16, 20, or 24 h. Experiments were carried out under dark conditions, apart from the illumination. Cells were viewed live to map intracellular drug localisation by detecting their natural fluorescence or they were fixed for Pgp immunofluorescence.

### Confocal microscopy for MTZ and hypericin localisation

Digital imaging of live cells to detect localisation of naturally fluorescent MTZ and hypericin was performed using a Zeiss confocal microscope (Carl Zeiss Ltd, Welyn Garden City, UK) equipped with a × 63 water immersion objective lens.

**Figure 3:**
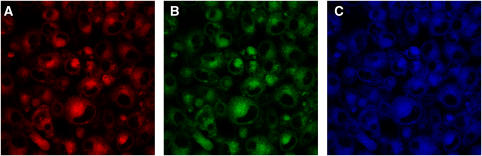
Cells treated with hypericin (HYP) and excited with 488 nm laser (16 h post-illumination). Detection of fluorescence at (**A**) 590 nm, (**B**) 660 nm and (**C**) 690 nm. The images highlight the highly fluorescent nature of HYP and the ‘spillover’ problem it presents when attempting to isolate its detection in one channel.

The strongly fluorescent hypericin often gives rise to ‘crosstalk’ when imaging in the presence of other fluorescent dyes, eg, organelle probes such as lysotracker, resulting in misleading observations (Theodossiou *et al*, 2004; Taroni *et al*, 2005). Therefore, we carried out fluorescence emission and excitation scans to determine the most ideal excitation and emission spectra for each drug with minimum or no overlap of emission in the two drugs' spectra upon detection. Three lasers were tested (488, 568, and 633 nm) to excite the specimens in all concurrent combinations and orders. Specifically, when hypericin-treated cells were excited with the 488 nm laser the main emission peak was at 595 nm with a secondary peak at 650 nm, but still detectable at 690 nm (where MTZ may be detected). Figure 3 highlights this ‘crosstalk’ effect of hypericin. When excited with the 633 nm laser, no fluorescence was detected at 660 nm and 690 nm. In turn, MTZ was found to have strong signals in two channels, particularly when excited with 633 nm. Of the two channels, 660 nm and 690 nm, the latter detected clearly both intranuclear and intracytoplasmic MTZ. We therefore decided to excite the two drugs separately with two different lasers; Specimens were exposed to a 488 nm laser to excite hypericin; emission was detected at 590 nm. Cells were then excited with the 633 nm laser for MTZ and detected at 690 nm. Images were overlaid for final combination images.

Additionally, lysotracker green DnD-26 (50 nM; Invitrogen Ltd, Paisley, UK) was added to some hypericin treated coverslips half an hour before illumination and was incubated at 37°C, to investigate co-localisation within lysosomes. For detection, cells were excited with a 488 nm laser (both agents); lysotracker emission was detected at 510 nm while hypericin was detected at 590 nm, with no crosstalk. The experiment was carried out with/ without illumination, and lysotracker only controls.

### Immunofluorescence staining for Pgp expression

P-glycoprotein expression was detected by indirect immunofluorescence, using a mouse anti-human monoclonal anti-Pgp antibody (IgG_1_) and a goat anti-mouse IgG1 secondary antibody (fluorescein-conjugated) (Oxford Biotechnology Ltd, Kidlington, Oxon, UK), detected by fluorescent microscopy (Carl Zeiss Ltd, Welyn Garden City, UK).

Cells were grown on 13 mm coverslips and treated with MTZ and/or hypericin as described (2.6). Coverslips were washed in PBS, fixed in acetone:methanol (50 : 50) (room temperature, 6 min), washed (3 × PBS) and stored (PBS, 4°C). For immunoflorescence, coverslips were incubated with the primary antibody (1 : 20 in PBS, 1 h, room temperature), washed in PBS (3 ×), incubated with the secondary antibody (1 : 50 in PBS, 1 h, 4^o^C, dark). After a final BPS wash (3 ×) coverslips were mounted onto slides using citifluor and stored at 4°C in the dark, till visualisation under the microscope.

Other fixing methods were inferior (100% acetone, paraformaldehyde, gluteraldehyde).

### Determination of Pgp function

To investigate whether hypericin impaired Pgp function, we compared it against verapamil, a known Pgp inhibitor. Pilot experiments determined that, in MGHU1/R cells, 24 h co-incubation of MTZ (0.1 *μ*g ml^−1^—which by itself kills <5% of resistant cells) with verapamil (0.1–10 *μ*M) reversed the resistant phenotype in a dose dependent manner (increased killing: <5% for 0.1 *μ*M verapamil; 18% increased killing for 1 *μ*M verapamil; 40% for 5 *μ*M; detailed results not shown). To compare the effect of hypericin on Pgp function with that of verapamil, we used the exact incubation conditions of hypericin and verapamil which increased killing (ie, 0.1 *μ*M HYP incubated for 4 h and verapamil incubated for 24 h) and investigated changes in Pgp efflux using Rhodamine123, a standard Pgp efflux agent. Our method is an adaptation of the seminal method for Pgp efflux assessment using flow cytometry described by Wang *et al* (2000, 2004).

Resistant cells were grown on 13 mm glass as above for 48 h. At 24 h cells received verapamil (0.1, 1, 10 *μ*M) for another 24 h or at 42 h cells received hypericin (0.1 *μ*M) for another 4 h (removal by washing, plus illumination). Cells were washed and Rhodamine123 (200 ng ml^−1^ in full medium) was ‘loaded’ onto the cells during a 30-min incubation (37°C, 5% CO_2_, humidity). Cells were washed immediately before confocal visualisation and left in PBS. Rhodamine123 was excited at 488 nm and fluorescent signal collected at 500–550 nm (no interference by hypericin crosstalk was detected). Images were taken every 30 s, for 20 min. Fluorescence uptake was heterogeneous in different cells with signal always localising in the cytoplasm. Changes in signal intensity over time was calculated for each cell by drawing cytoplasmic regions of interest (in approximately eight cells per coverslip) and calculating the per cent decrease in intensity for each cell after 20 min of efflux.

### Statistical analysis

Single agent and combination viability experiments were repeated 6–12 times. Raw data underwent one-way ANOVA followed by appropriate *post hoc* analysis, Tukeys (multiple simultaneous comparisons), or Dunnets (dose–response curves for single agents). When all light *vs* all dark data were compared (to identify differences in the killing effect of illuminated and dark HYP+MTZ combinations), the dark control data was consistently slightly lower than the light control data (<10%) and the former was normalised based on the difference of means of the two untreated control groups. Analysis was carried out on normalised data by one-way ANOVA followed by Bonferroni's multiple comparisons test, which takes into account unequal group sizes. Although data are parametric and were analysed statistically as such, the majority are depicted graphically or described in terms of percentages, for clarity. Confocal experiments were repeated a minimum of eight times. Pgp efflux confocal measurements were also parametric, however the uptake of fluorescent Rhodamine123 was heterogeneous and the resultant decrease values had a fairly wide range, unlike the experiments carried out in 96-well plates. Therefore, the data was submitted to Mann–Whitney non-parametric analysis.

## RESULTS

### Mitoxantrone and hypericin dose–response curves

#### Mitoxantrone

Cell viability was assessed after MTZ incubation followed by a 24 h recovery period. Killing rates of approximately 6 and 28% were detected for MGHU1/R and 80 and 89% were detected for MGHU1/S for 0.1 and 0.2 *μ*g ml^−1^ MTZ, respectively. By 2 *μ*g ml^−1^ of MTZ, killing was increased to 60% for resistant cells and 95% for sensitive cells. In contrast, both MCF7 cell lines were more resistant to low doses of MTZ than MGHU1 cell lines. MTZ at 0.1 and 0.2 *μ*g ml^−1^ killed 6 and 13% of MCF-7/R and 16 and 26% of MCF-7/S. By 2 *μ*g ml^−1^, MTZ killed 60 and 83% of resistant and sensitive cells respectively. MTZ doses chosen to be used for combination experiments were 0.1 and 0.2 *μ*g ml^−1^; with these doses cell viability is still evident and therefore further added toxicity from hypericin can be detected.

#### Hypericin

Cells were incubated with hypericin for 4 h, before illumination with blue light (1, 2, or 5 min); cell viability was assessed 24 h later. Generally, the longer the duration of light, the more evident the phototoxic effect of hypericin at a lower dose: Using MGHU1/R as an example, with 1 min illumination hypericin had evident phototoxic effects from 0.4 *μ*M. (viability reduced to 60% of control); with 2 min illumination hypericin had effects from 0.2 *μ*M (viability reduced to 64%); and with 5 min illumination hypericin effects were evident at 0.1 *μ*M (viability reduced to 21.2%).

**Figure 4:**
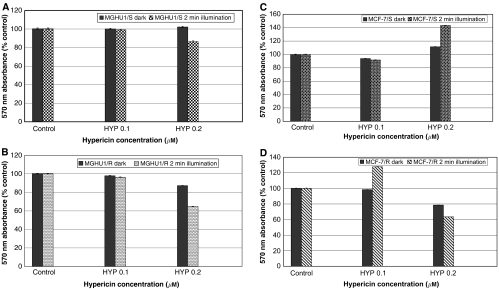
Bladder cancer cells (**A**) MGHU1/S and (**B**) MGHU1/R and breast cancer cells (**C**) MCF-7/S and (**D**) MCF-7/R were incubated with hypericin (HYP, 0.1 *μ*M–0.2 *μ*M) for 4 h, washed and experimental groups were illuminated for 2 min with blue light while dark controls were not exposed to light. Cells were allowed to recover for a further 24 h and were subjected to the MTT assay. Absorbance at 570 nm corresponds to viability and was expressed as a percentage of absorbance by untreated cells (*y* axis). *P*<0.05 for: MGHU1/R light *vs* dark, 0.2 *μ*M HYP; MCF-7/S light *vs* dark, 0.2 *μ*M HYP; MCF-7/S light *vs* dark, 0.1 *μ*M HYP.

Generally, parental cell lines were less susceptible to hypericin than their MDR counterparts. Figures 4A and B illustrate ‘dark’ and light activated (2 min) toxicity of hypericin in MGHU1/R and MGHU1/S cells. Unlike the pattern in the drug-sensitive cells, the resistant cells exhibited some ‘dark’ cytotoxicity to hypericin (0.1 *μ*M, 0.2 *μ*M). Cellular killing was on average 13% more in the illuminated MDR cells compared to ‘dark’ controls (*P*<0.05), demonstrating direct phototoxicity. An interesting observation was made with the MCF-7 cell lines: Both MCF-7R and MCF-7/S cells appeared to proliferate in response to hypericin plus illumination (*P*<0.05 light *vs* dark, MCF-7/S at 0.2 *μ*M HYP; MCF-7/R at 0.1 *μ*M HYP). At higher doses, cells succumbed to the toxic effects of hypericin plus illumination, with MCF-7/S cells being susceptible from 0.4 *μ*M (not shown) and MCF-7/R from 0.2 *μ*M of hypericin. Similarly to MGHU1/R, MCF-7/R exhibited some dark cytotoxicity (Figure 4C and D). The differences observed between the four cell lines appear to be independent of the level of hypericin uptake as shown in the level of fluorescence emitted at 605 nm. There was no significant difference in initial hypericin uptake, with an average of 3.6–3.9 (fluorescence emitted) across all four cell lines.

Hypericin doses chosen to be used in the combination experiments were 0.1 and 0.2 *μ*M. At these concentrations the killing effect (with or without illumination) is minimal or non-existent, thus it is possible to determine if hypericin has an additive and/or synergistic effect when combined with MTZ. The duration of illumination chosen for the combination experiments was 2 min, which is a compromise between minimal level of cytotoxicity in the MGHU1 cell lines and reducing the apparent phenomenon of cellular proliferation, at lower doses, in the MCF-7 cell lines.

### Mitoxantrone and hypericin combination effects on cell viability

Cells were incubated with MTZ for 20 h, then a combination of hypericin and MTZ (4 h), before being illuminated (2 min). Viability was assessed 24 h later.

**Figure 5:**
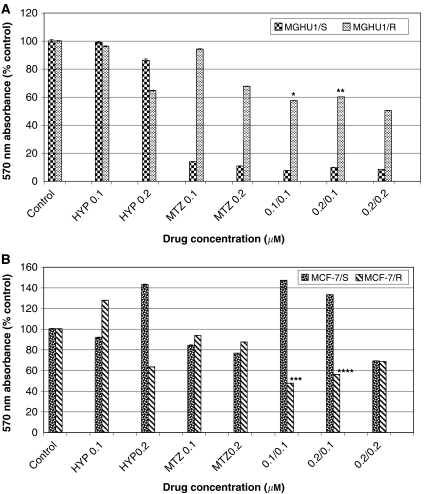
Bladder cancer cells (**A**) MGHU1/S and MGHU1/R and breast cancer cells (**B**) MCF-7/S and MCF-7/R were incubated with hypericin alone (HYP, 0.1 or 0.2 *μ*M) for 4 h or with mitoxantrone (MTZ, 0.1 or 0.2 *μ*g ml^−1^) alone for 24 h or with mitoxantrone (0.1 or 0.2 *μ*g ml^−1^) for 20 h, followed by 4 h incubation with mitoxantrone+hypericin (MTZ 0.1 or 0.2 *μ*g ml^−1^+HYP 0.1 *μ*M or 0.2 *μ*M) and illuminated (2 min) with blue light (on the combination groups, the first number denotes the HYP concentration; the second number denotes the MTZ concentration). Cells were allowed to recover for a further 24 h and were subjected to the MTT assay. Absorbance at 570 nm corresponds to viability and was expressed as a percentage of absorbance by untreated cells (*y* axis). ^*^38.65%, 36.63% increased killing *vs* hypericin or MTZ alone, respectively, *P*<0.05; ^**^34% increased killing *vs* MTZ alone, *P*<0.05; ^***^80.2, 46.1% increased killing *vs* hypericin or MTZ alone, respectively, *P*<0.001; ^****^37.6% increased killing *vs* MTZ alone, *P*<0.01.

MGHU1/R cells exhibited increased killing when exposed to the hypericin+MTZ combination, compared to hypericin or MTZ alone (Figure 5A). There was a 38.65 and 36.63% decrease in cell viability of MGHU1/R cells when exposed to hypericin (0.1 *μ*M)+MTZ (0.1 *μ*g ml^−1^) combination, compared to hypericin (PDT) or MTZ alone (*P*<0.05), respectively (Figure 5A). There was also a significant decrease in cell viability (by 34%) of MGHU1/R cells when exposed to hypericin (0.2 *μ*M)+MTZ (0.1 *μ*g ml^−1^) combination, compared to MTZ alone (*P*<0.05) (Figure 5A). Similarly, MCF-7/R cells showed significantly increased killing (80.20%, *P*<0.001; 46.10%, *P*<0.001) with the hypericin (0.1 *μ*M)+MTZ (0.1 *μ*g ml^−1^) combination, compared to hypericin or MTZ alone, respectively. MCF-7/R cells also exhibited significantly increased cell killing, 37.60% decrease in cell viability (*P*<0.01) (Figure 5B), with the hypericin (0.2 *μ*M)+MTZ (0.1 *μ*g ml^−1^) combination compared to MTZ alone.

**Figure 6:**
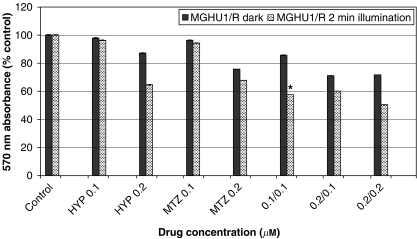
Viability of MDR bladder cancer cells under light and dark conditions. Cells were incubated with hypericin alone (HYP, 0.1 or 0.2 *μ*M) for 4 h or with mitoxantrone alone (MTZ, 0.1 or 0.2 *μ*g ml^−1^) for 24 h or with mitoxantrone (0.1 or 0.2 *μ*g ml^−1^) for 20 h, followed by 4 h incubation with mitoxantrone+hypericin (MTZ 0.1 or 0.2 *μ*g ml^−1^+HYP 0.1 *μ*M or 0.2 *μ*M) and illuminated (2 min) with blue light (on the combination groups, the first number denotes the HYP concentration; the second number denotes the MTZ concentration). Parallel experiments were conducted in the dark. Cells were allowed to recover for a further 24 h and were subjected to the MTT assay. Absorbance at 570 nm corresponds to viability and was expressed as a percentage of absorbance by untreated cells (*y* axis). Statistical significance presented only for dark *vs* light combinations. ^*^28.15% increased killing light *vs* dark, *P*<0.05.

Dark control plates were run concurrently to investigate the effect of illumination on viability when cells were treated with both hypericin and MTZ. Results are presented in a composite graph (Figure 6). MGHU1/R cells which were exposed to combinations of hypericin and MTZ (0.1 *μ*M+0.1 *μ*g ml^−1^) and illuminated showed significantly more killing than their dark counterparts (28.15%, *P*<0.05). Although there were also differences between light and dark in the other two drug combinations, they did not quite reach significance. Similar results were found for MCF-7/R cells (not shown).

### Intracellular imaging of mitoxantrone and hypericin

**Figure 7:**
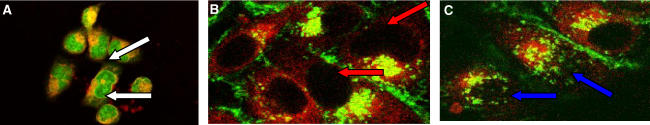
(**A**) MGHU1/S cells (low magnification), (**B**) and (**C**) MGHU1/R cells treated with 2 *μ*g ml^−1^ MTZ for 20 h followed by 4 h of incubation with 2 *μ*g ml^−1^ MTZ+2 *μ*M HYP. Specimens (**A**) and (**C**) were illuminated for 2 min, specimen (**B**) is a dark control. Images were taken at 1 h post-illumination. The white arrows indicate nuclear MTZ in the MGHU1/S cell line. The red arrows show the nuclei of MGHU1/R cells (dark control) with no nuclear MTZ localisation. The blue arrows show nuclear MTZ in some MGHU1/R cells (illuminated). (MTZ=green; HYP=red; co-localisation=yellow).

Cells were incubated with drugs either alone or in combination for the desired time and illuminated. Cells were left to recover for different time points (from 1 to 24 h) and typical images are presented here. Throughout all imaging experiments hypericin was localised only to the cytoplasm, whereas MTZ was also found in the nucleus of sensitive cells or some illuminated MDR cells. Where co-localisation was observed, the two agents often appeared to co-exist as cytoplasmic vesicles. Early after illumination (1 h), all cell lines showed some cytoplasmic co-localisation of hypericin and MTZ, as illustrated in Figure 7. At this time, both drug-sensitive cell lines showed clear nuclear localisation of MTZ as expected; illumination does not alter this pattern (Figure 7A). At the same time MDR cell lines, which were not illuminated (dark controls), showed no nuclear MTZ localisation, which is typical of Pgp-overexpressing cells (Figure 7B). Interestingly, the pattern of green fluorescence changed when resistant cells were illuminated, with MTZ appearing to re-localise nearer to or within the nucleus of some, but not all, cells. Hypericin localisation, which was evident throughout the cytoplasm, did not appear to change on illumination (Figure 7C). Parallel experiments where MDR cells were treated with MTZ alone (and illuminated) did not show any nuclear drug localisation (image not shown).

**Figure 8:**
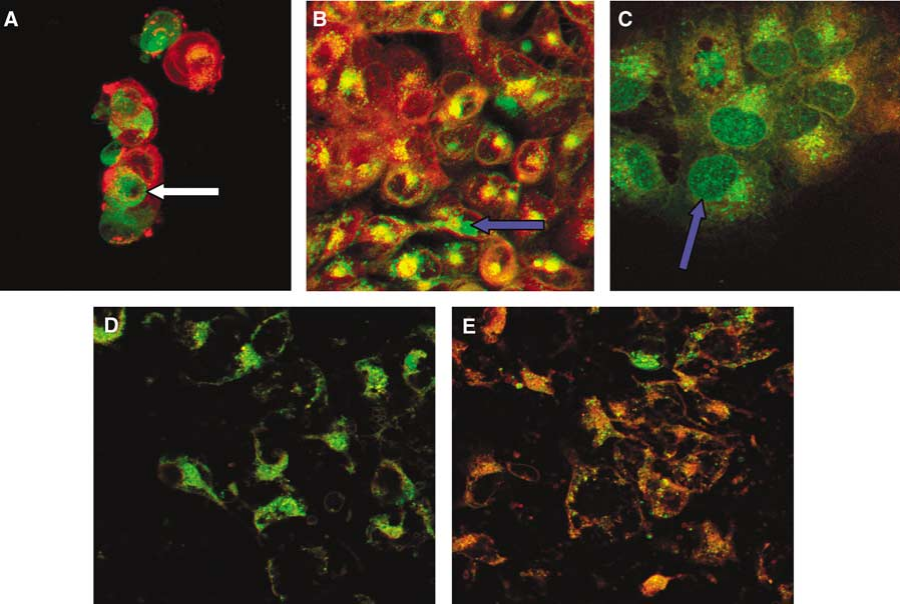
Cells were treated with 2 *μ*g ml^−1^ MTZ for 20 h followed by 4 h of incubation with 2 *μ*g ml^−1^ MTZ+2 *μ*M HYP. Specimens were then illuminated for 2 min. Images were taken post-illumination at 4 h for (**A**) MCF-7/S and (**B**) MCF-7/R; at 16 h for (**C**) MCF-7/R; at 24 h for (**D**) MGHU1/S and (**E**) MGHU1/R. The white arrow (**A**) shows nuclear MTZ in an MCF-7/S cell. The blue arrows show nuclear localisation in MCF-7/R cells at 4 h (**B**) and 16 h (**C**) post-illumination. At 24 h (**D**, **E**) MGHU1/R cells appear to have more red fluorescence than MGHU1/S cells. (MTZ=green; HYP=red; co-localisation=yellow).

Figure 8 shows MCF-7 cell lines treated with both drugs, 4 h after illumination. Again there was clear nuclear MTZ localisation in MCF-7/S cells, as expected (Figure 8A). In the equivalent illuminated MCF-7/R cells (Figure 8B) there was MTZ present in the nucleus of some cells. There was clearer co-localisation of the drugs compared to the sensitive cells. At 16 h post-illumination, the intranuclear MTZ fluorescence increased in some MDR cells, with a striking example of MCF-7/R cells shown in Figure 8C. The same patterns of drug and photosensitiser localisation over time were seen for MGHU1 cell lines.

Images at 24 h post-illumination showed more sparsely distributed cultures with cells appearing stressed, especially sensitive cells. Figure 8D is an image of MGHU1/S cells. Here there was lack of significant localisation of MTZ to the nucleus. This is not surprising, given that the majority of cells in these cultures would have been successfully assaulted by treatment and had already died. The remaining cells imaged here must have evaded MTZ-mediated killing. The equivalent MGHU1/R cells (Figure 8E) show increased hypericin retention compared to the parental cells and this is a recurrent feature for resistant cells by 24 h.

**Figure 9:**
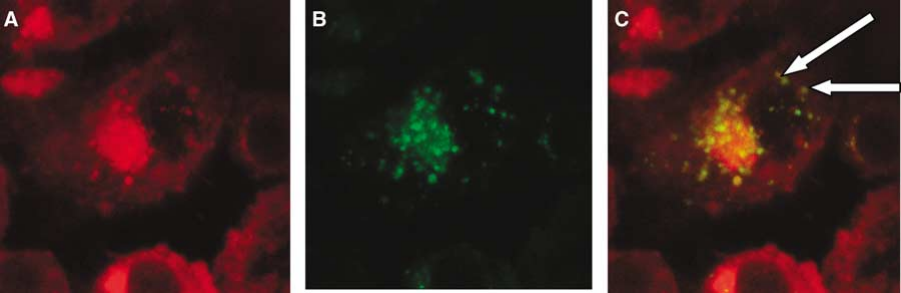
MGHU1/R cells treated with HYP 2 *μ*M according to the described timeline. Lysotracker green DND-26 (50 nM) was added for half an hour (end of incubation) (dark experiment). (**A**) hypericin signal, (**B**) lysotracker green DND-26, (**C**) overlay, showing co-localisation. White arrows point out examples of areas of co-localisation. (hypericin=red, Lysotracker green DND-26=green, co-localisation=yellow).

Co-incubation experiments with lysotracker green DND-26 showed some localisation between hypericin and lysotracker in cytoplasmic vesicles (lysosomes, Figure 9). Illumination resulted in some relocalisation of both lysotracker and hypericin (time lapse observation 10 min for lysotracker (signal bleached), 20 min for hypericin), without the signal disappearing dramatically or dispersing.

### Pgp expression on the addition of hypericin and mitoxantrone

**Figure 10:**
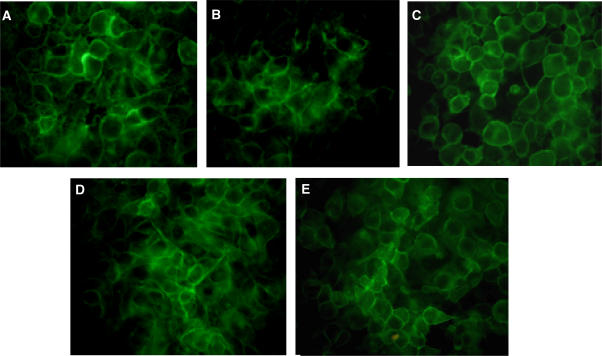
MGHU1/R cells treated with (**A**, **D**, **E**) 2 *μ*M HYP, (**B**) 2 *μ*g ml^−1^ MTZ and (**C**) 2 *μ*g ml^−1^ MTZ+2 *μ*M HYP, according to the described timeline. Cells (**A**, **B**, **C**) were illuminated for 2 min and the experiment was terminated 4 h post-illumination. To determine the effect of light, cells were either illuminated for 2 min (**E**) or were kept as dark controls (**D**) and the experiment terminated at 24 h. P-glycoprotein, detected by indirect immunofluorescence (white), is situated mostly in the cellular membrane.

We used indirect immunofluorescence to investigate any changes in Pgp expression upon the addition of the drugs, either alone or in combination, over a 24 h period. Overall, Pgp immunoflorescence showed no difference in protein expression, in either illuminated specimens or in dark controls when imaged at a variety of time intervals up to and including 24 h post-illumination. Figure 10 shows the results of Pgp staining on the MGHU1/R cell line treated with (1) hypericin, (2) MTZ, and (3) both agents. Visually, the Pgp immunofluorescence is very similar, regardless of drug exposure. Results for MCF-7/R were similar (not shown). Furthermore, there was no apparent difference in Pgp expression between dark or illuminated slides, at any time post-incubation either (Figure 10D and E). Immunofluorescence of the sensitive parental cell lines showed virtually no Pgp expression and therefore no further drug incubation experiments were carried out in these cell lines.

### Pgp function

The integrity of Pgp function was investigated by measuring Pgp-mediated Rhodamine123 efflux over 20 min on confocal microscopy, after the resistant cells were pre-incubated either with hypericin or increasing concentrations of verapamil, the classical Pgp inhibitor. Hypericin pre-incubated cells lost 74% of fluorescent Rhodamine123 signal after 20 min of efflux compared to initial amounts taken up (Table 1). This was similar to control untreated cells, which lost 72.4%. In contrast, verapamil pre-incubated cells lost fluorescence in a dose-related manner: the cells which received the smallest verapamil concentration (0.1 *μ*M) lost 35.4% of signal, for 1.0 *μ*M verapamil the decrease was 20%, while for 10 *μ*M verapamil the decrease was 13.5%. For the latter group, the range (−14.8 to 25.9) indicates that during general efflux some cells were taking up and retaining Rhodamine123, which was present in their immediate environment, presumably effluxed by neighbouring cells. Per cent decrease values for all verapamil doses were significantly different from controls (*P*<0.01, Mann–Whitney).

## DISCUSSION

### Hypericin-induced photodynamic therapy

The first aim of this study was to determine the doses and duration of illumination at which hypericin (PDT) resulted in minimal or no killing for both resistant cell lines. Previous PDT studies on the effect of hypericin on cultured fibroblasts or murine keratinocytes have shown concentration- and light-dependent phototoxicity starting at 1.25 or 5 *μ*M, depending on cell type. The main line of investigation of the light dependency of hypericin-induced PDT on murine keratinocytes had centred on varying doses (joules) of light (0.1–10 J cm^−2^) (Yu *et al*, 1996; Theodossiou *et al*, 2004). We observed the effect of a concentration range of hypericin (0.1–1.0 *μ*M) over light exposure of 1, 2, or 5 min. Generally, the paternal drug-sensitive cell lines were more resistant to hypericin than their MDR counterparts. A concentration- and light duration-dependent cell killing of the MGHU1 cell lines was observed, with longer exposure and higher concentrations resulting in higher toxicity, in line with previous work in the field (Adreoni *et al*, 1994; Yu *et al*, 1996; Kamuhabwa *et al*, 2004; Theodossiou *et al*, 2004). Hypericin at 0.2 *μ*M was the lowest concentration at our chosen duration of illumination (2 min), which resulted in significant cytotoxicity in MGHU1/R cells, but this dose did not affect MGHU1/S growth.

The MCF-7 cell lines, exhibited a more complex response, succumbing to phototoxic effects at higher hypericin doses (from 0.4 *μ*M for sensitive cells and from 0.2 *μ*M for resistant cells, 2 min illumination), but exhibiting apparent proliferation at lower doses. Previous reports show that, in response to physical or chemical stress, cells can activate signalling pathways, including the mitogen-activated protein kinase (MAPK) and phosphotidylinositol-3-kinase pathways (Oleinick and Evans, 1998; Moor, 2000; Agostinis *et al*, 2002). Specifically in HeLa cells photosensitisation with hypericin resulted in the rapid and sustained activation of Jun N-terminal kinase-1 and MAPK (Assefa *et al*, 1999). These molecules are capable of propagating a proliferation signal and this may be the mechanism triggered in the MCF-7 cell lines in response to low-dose hypericin photosensitisation. The differences observed between the four cell lines appear to be independent of the level of hypericin uptake as shown the level of fluorescence emitted at 605 nm. There was no significant difference in initial hypericin uptake, with an average fluorescence intensity of 3.6–3.9 (arbitrary units) across all four cell lines.

### Photochemical internalisation

The potentiating effects of PCI have previously been demonstrated *in vitro* (Engesaeter *et al*, 2006) and *in vivo* (Selbo *et al*, 2001; Dietz *et al*, 2005) and these results have been reviewed by Høgset (Høgset *et al*, 2004). While most studies have been carried out using amphiphilic photosensitisers (eg, TPPS_2a_) PCI has also been demonstrated using a membrane localising sensitiser, ALA-induced photoporphyrin IX. Selbo *et al*, 2001 administered the photosensitiser aluminium disulphonated phthalocyanine by intraperitoneal injection into athymic mice, followed (48 h later) by a single intratumoural injection of the protein toxin gelonin. The findings indicated that the photosensitiser could become localised in endosomes, *in vivo*, within the tumour cells and that upon illumination it could be relocalised to the cytoplasm. Furthermore, the treatment regime demonstrated that PCI of gelonin significantly potentiated its inhibition of tumour growth: 67% of mice administered PCI treatment became completely tumour free, compared to 10% of those treated with PDT alone, and none of those treated with gelonin alone.

In this study, illumination of MDR cancer cells which had been pre-incubated with a combination of the photosensitiser hypericin and the chemotherapeutic MTZ resulted in increased killing compared to unilluminated dark controls. The effect was pronounced at low concentrations of the two agents, 0.1 *μ*M hypericin and 0.1 *μ*g ml^−1^ MTZ, using a 2 min illumination period. These particular concentrations of hypericin and MTZ were not significantly cytotoxic when cells were exposed to each agent alone (with or without light); on the contrary hypericin, when photoactivated, appeared to stimulate MCF-7/R proliferation. Furthermore, under light conditions (Figure 5A and B), there was a 38.65% increase in killing of MGHU1/R cells when the drugs were combined and illuminated compared to cells that received PDT alone (ie, hypericin plus illumination) and a 36.63% increase compared to MTZ alone. A similar pattern was observed in MCF-7/R cells. These findings are consistent with PCI. Consequently, by potentiating the cytotoxicity of MTZ, hypericin plus illumination appear to circumvent the MDR phenotype; since hypericin by itself at the doses used does not affect cellular viability, the combined effect is probably due to synergism. Furthermore, hypericin potentiated the killing activity of MTZ on the sensitive cell lines but this was minimal and not significant. Similarly, Canti *et al* (1998) demonstrated that when antiblastic drugs, ADR and cisplatinum (CDDP) were combined with the photosensitiser aluminium disulphonated phthalocyanine it resulted in a significant additive effect on the killing of murine leukaemia and lymphoma lines, compared to ADR or CDDP alone.

Confocal imaging experiments explored the intracellular localisation of the two agents. The findings indicate that hypericin and MTZ colocalised in cytoplasmic vesicles of both sensitive and MDR cells both prior and following illumination. MTZ was present in the nucleus of sensitive cells, as expected, at all experimental end-times apart from those cells that survived the cytotoxic insult at 24 h. MDR cells when not illuminated excluded MTZ from the nucleus (in the presence or absence of hypericin). However, when illuminated in the presence of hypericin, some MTZ became nuclear (4 and 16 h post-illumination) and this was apparent in a proportion of cells. Hypericin was cytoplasmic throughout and appeared to be retained better by resistant cells compared to sensitive cells at 24 h (compared to the initial uptake of hypericin which appeared equivalent in all cell lines, as determined by fluorescent plate reading). The distribution of hypericin was similar for dark and illuminated cells in our experiments. Furthermore, as reported in previous work by colleagues, we showed hypericin partly co-localising with lysotracker green DND-26, a marker known to bind to acidic membranes (eg, lysosomal vesicles) (Theodossiou *et al*, 2004). This work is supplemented by another study which highlighted that the presence of serum during hypericin incubation favours endocytic uptake (Siboni *et al*, 2002). We also showed co-localisation between hypericin and MTZ, the latter may be taken up by endocytosis and is retained inpart by lysosomes. Furthermore, following illumination, part of the MTZ fluorescence appears to move towards and into the nucleus of resistant cells. This suggests that co-localisation takes place in endolysosomal compartments and that illumination liberates MTZ, consequently facilitating the drug reaching its nuclear target. Since MTZ is a weak base, then liberation from an acidic vesicle, such as a lysosome (where MTZ is ‘ion-trapped’) would increase the availability of this drug to intracellular targets (Mahoney *et al*, 2003). The fact that the hypericin signal did not undergo major changes upon illumination is not surprising since in an effort to detect clear fluorescent signals the amounts used for our confocal experiments were 10-fold greater than those used for the cell viability experiments. Consequently, hypericin was detected throughout the cell cytoplasm. Lou *et al* (2006) also showed nuclear localisation of MTZ in MDR cells, however the underlying mechanisms differ in that the photosensitiser (plus illumination) was administered before chemotherapeutic insult. The latter probably resulted in the destruction of endosomes and therefore their unavailability to transport chemotherapeutic drug (Prasmickaite *et al*, 2002).

Whether increased killing effects were the result of changes in Pgp efflux pump expression or function due to the addition of hypericin was also investigated. The addition of either agent by itself or in combination (+/− illumination) resulted in no changes in Pgp expression by MDR cell lines, as shown by immunofluorescent staining. Efflux of Rhodamine123 via Pgp pumps was detected by confocal microscopy. The method we used was based on the seminal work by Wang *et al* (2000) where the authors presented a flow cytometric method for investigating inhibition of Pgp efflux. In our work, cells which were pre-incubated with 0.1 *μ*M hypericin (plus illumination) did not interfere with Rhodamine123 efflux, unlike cells pre-incubated with verapamil (0.1 to 10 *μ*M). Doses for both agents were those that resulted in increased killing in ‘drug-combination’ experiments, therefore the amounts of both agents were pharmacologically active in our model system. The concentrations of agents used are of prime consideration since previous reports have presented superficially conflicting results on the action of St John's Wort constituents (hyperforin and hypericin) on Pgp expression and function. St John's Wort may alter the expression of various membrane pumps and result in decreased bioavailability of a variety of drugs. Hypericin at a concentration of 10 *μ*M induced mRNA expression of the Pgp gene MRD1 in the human colorectal carcinoma line LS180 (Gutmann *et al*, 2006). In contrast, Tian *et al* (2005) reported that only hyperforin and not hypericin (at 0.1 *μ*M, 24–48 h treatment) caused a time-/dose-dependent induction of Pgp protein by the same cell line. Furthermore, hypericin at the same concentration did not interfere with either digoxin efflux or Pgp-mediated transcellular transport of digoxin. However, Wang and his colleagues (2004) demonstrated that hypericin inhibited Pgp-mediated efflux at high concentrations (10 *μ*M).

The present study demonstrates that the cytotoxic efficacy of MTZ, against MDR cancer cell lines is increased when in combination with hypericin under illuminated (light) compared to dark conditions. Our experiments indicate that the two drugs do co-localise within the cytoplasm and that upon illumination some MTZ is mobilised to the nucleus. These observations appear to be independent of any effect on Pgp expression and/or function and are not mirrored under dark conditions. Taken collectively the findings of this study are consistent with PCI. PCI has the potential to have a wide impact in clinical practice, including (1) reducing the systemic side effects of anti-cancer cytotoxic agents, for two reasons, first by reducing the dose of drug required for a given killing effect and second by conferring an increased level of treatment selectivity as the cytotoxic effect will be greatest in areas that are illuminated and minimised systemically; (2) widening the therapeutic index of anti-cancer agents giving clinicians and patients a wider scope of drug dosing and effect; (3) reversing MDR, thus overcoming the major limiting factor in conventional anti-cancer chemotherapy regimens; and (4) providing a potential delivery mechanism for gene therapy (Høgset *et al*, 2004; Engesaeter *et al*, 2006). Thus this potentially versatile technique may provide a driving force for improving the delivery mechanisms in many drug-mediated treatment regimens.

## Figures and Tables

**Figure 1:**
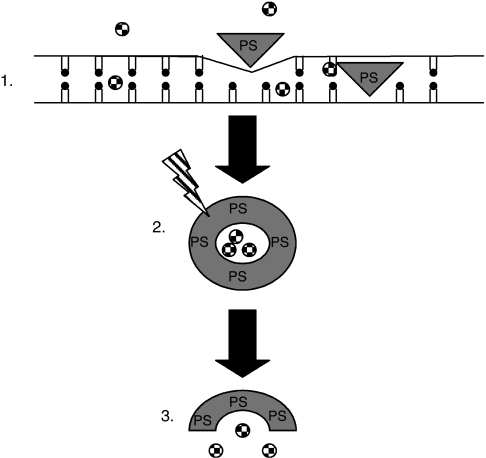
Diagrammatic representation of PCI. (1) Photosensitiser (PS) and cytotoxic drug (
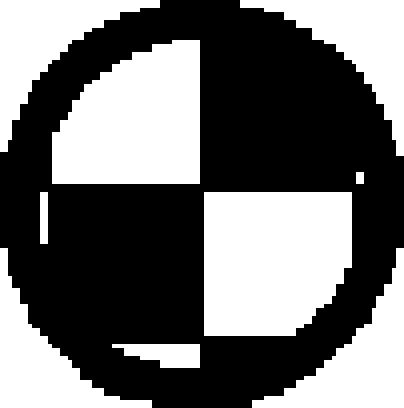
) incorporate into the inner layer of the phospholipid membrane of the target cell. (2) Endocytosed PS preferentially localises to the endocytic vesicular membrane with cytotoxic drug situated in the lumen. Upon irradiation with light of a specific wavelength the photosensitiser is excited, which in the presence of molecular oxygen produces reactive oxygen species, singlet oxygen being of particular importance. (3) These degrade and rupture the membrane releasing the cytotoxic drug directly into the cytosol.

**Table 1:** Rhodamine Efflux from MGHU1/R Cells

**Treatment**	**RD efflux (% decrease), 20 min median (range)**
VER 0.1	35.4 (28.8–50.4)
VER 1.0	20.0 (16.7–35.2)
VER 10	13.6 (−14.8–25.9)
HYP 0.1	74.0 (58.8–94.8)
Control	72.4 (58.9–81.5)

Experimental cultures were preincubated with verapamil (VER, 0.1, 1.0, or 10 *μ*M, 24 h) or hypericin (HYP, 0.1 *μ*M, 4 h, plus illumination). Cells were loaded with Rhodamine123 (30 min) and efflux semi-quantitated by confocal imaging, using 30 s slices, 20 min.

Per cent decrease refers to signal detected after 20 min efflux compared to signal at the beginning of efflux.

Between 5 and 8 cells per coverslip were calculated and results presented as medians (range).

Rhodamine123 was excited at 488 nm and emission spectra detected at 510–550 nm.

Per cent decrease values for all verapamil doses were significantly different from controls (*P*<0.01, Mann–Whitney).
